# Supplementary material for: Dissecting heterogeneity in malignant pleural mesothelioma through histo-molecular gradients for clinical applications
Source: Nat Commun. 2019 Mar 22;10:1333. doi: 10.1038/s41467-019-09307-6 (PMC6430832; doi:10.1038/s41467-019-09307-6)
Supplement: Supplementary file 1 — Supplementary Information [file 41467_2019_9307_MOESM1_ESM.pdf]

SUPPLEMENTARY INFORMATION

Dissecting heterogeneity in malignant pleural mesothelioma through histo-molecular gradients for clinical applications

Blum, *et al.*

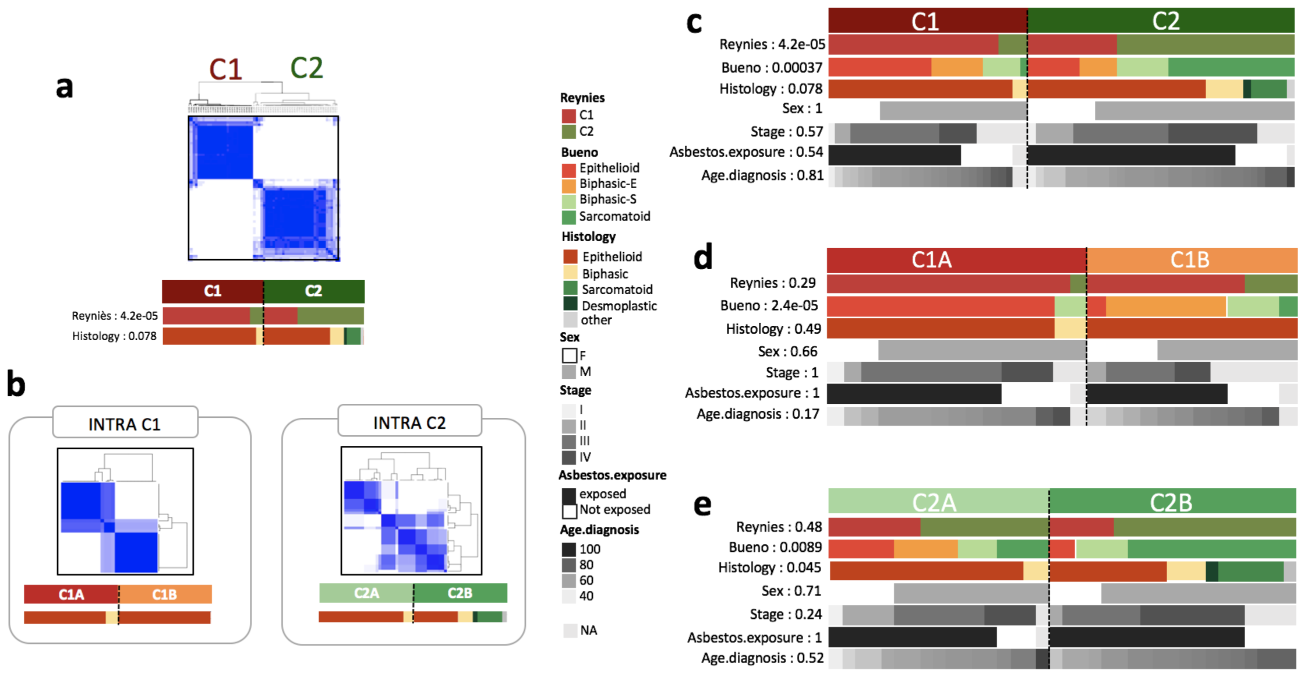

Supplementary Figure 1

Unsupervised clustering and subtype association with clinical annotations, histology and published classifications

a Dendrogram and co-classification matrix resulting from consensus clustering analysis based on transcriptomic data of 63 tumor samples. The strength of the blue color is proportional to the frequency at which samples have been clustered together. The Fisher's exact *P* values show the association between C1/C2 clustering and the centroid-based prediction of Reynies MPM subtypes and the histology. **b** Co-classification matrix of intra-C1 or intra-C2 samples. Histology visualization (bottom) shows that MMS samples are exclusively in C2B subtype. **c,d,e** The figures show for the C1/C2 subtypes (**c**), the C1A/C1B subtypes (**d**), the C2A/C2B subtypes (**e**), the prediction of MPM subtypes using transcriptome publically available classifications (Reynies, Bueno) and their clinical and histopathological characteristics (histology, sex, stage of the disease, asbestos exposure and age at diagnosis). *P* values of Fisher's exact test are indicated to show the association of each feature with the classifications.

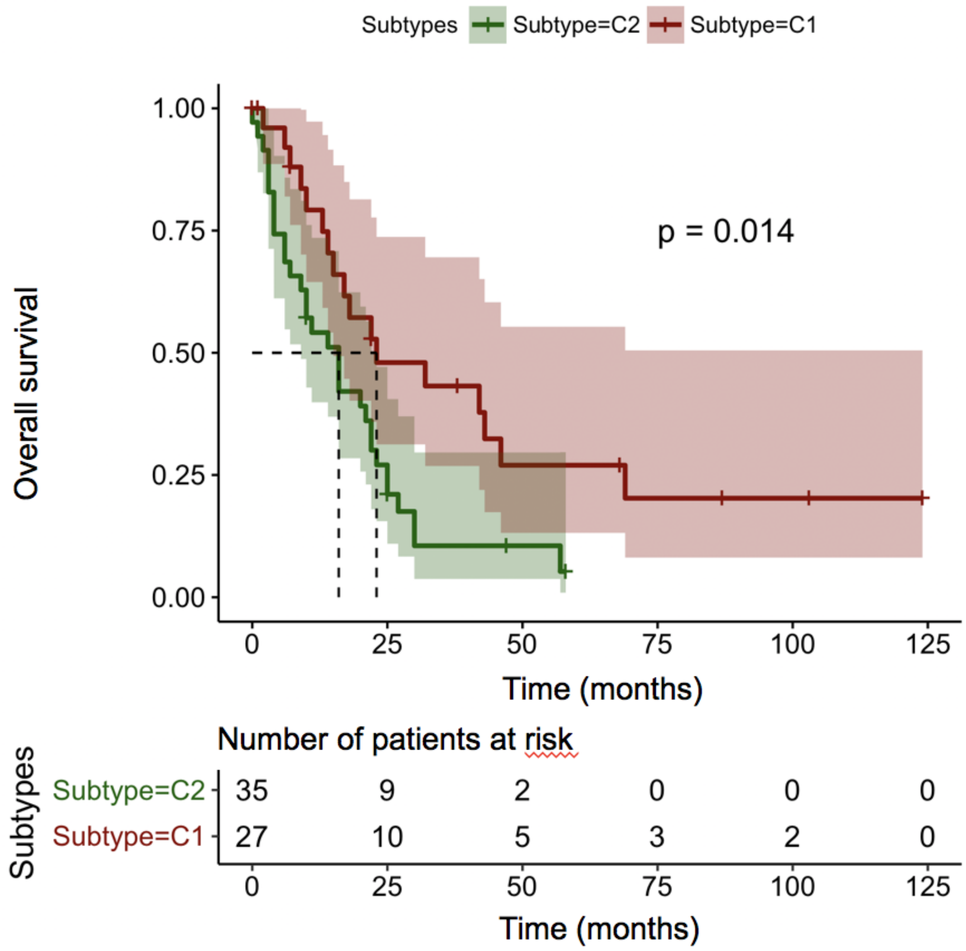

Supplementary Figure 2

Overall survival differences between C1 and C2 MPM subtypes.

Overall survival curves of patients related to C1 (red) and C2 (green) molecular subtypes. Survival curves were estimated using the Kaplan-Meier method and compared with the log-rank test.

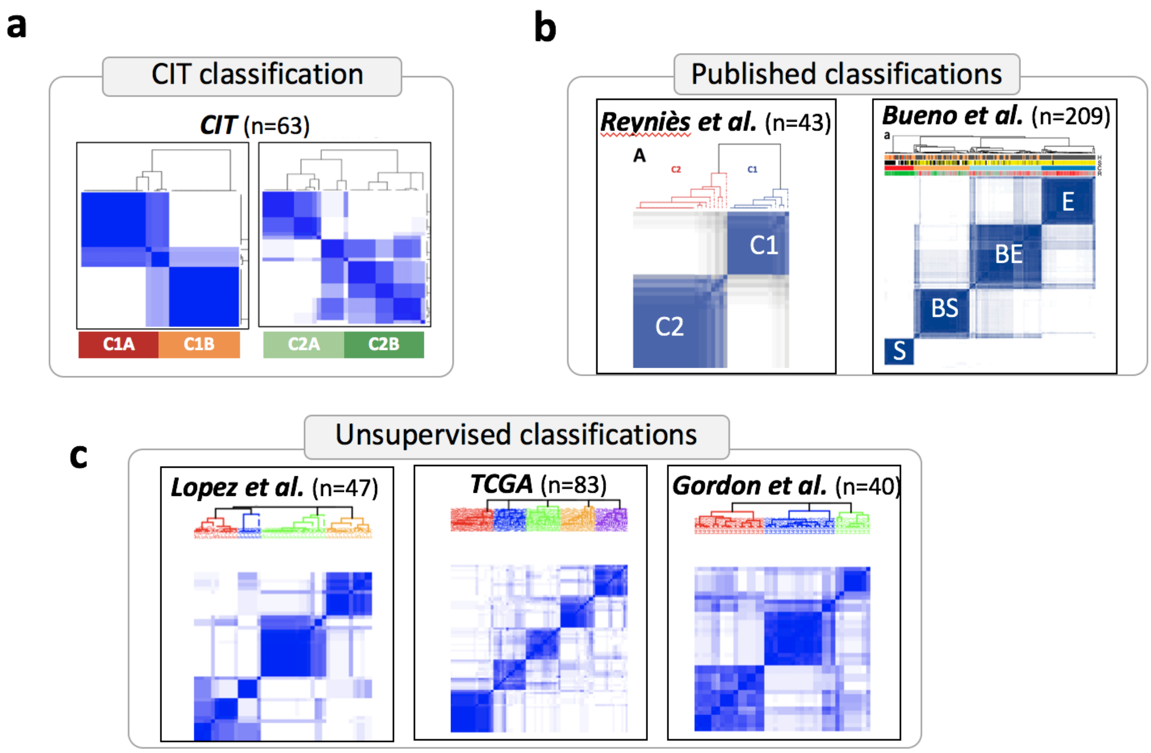

Supplementary Figure 3

Meta-analysis comparing clustering from different datasets.

Dendrograms and co-classification matrices resulting from consensus clustering analysis based on transcriptomic profiles from our dataset (CIT) (**a**), from published studies (Reynies, Bueno) (**b**) and from other public datasets (Lopez, TCGA, Gordon) (**c**).

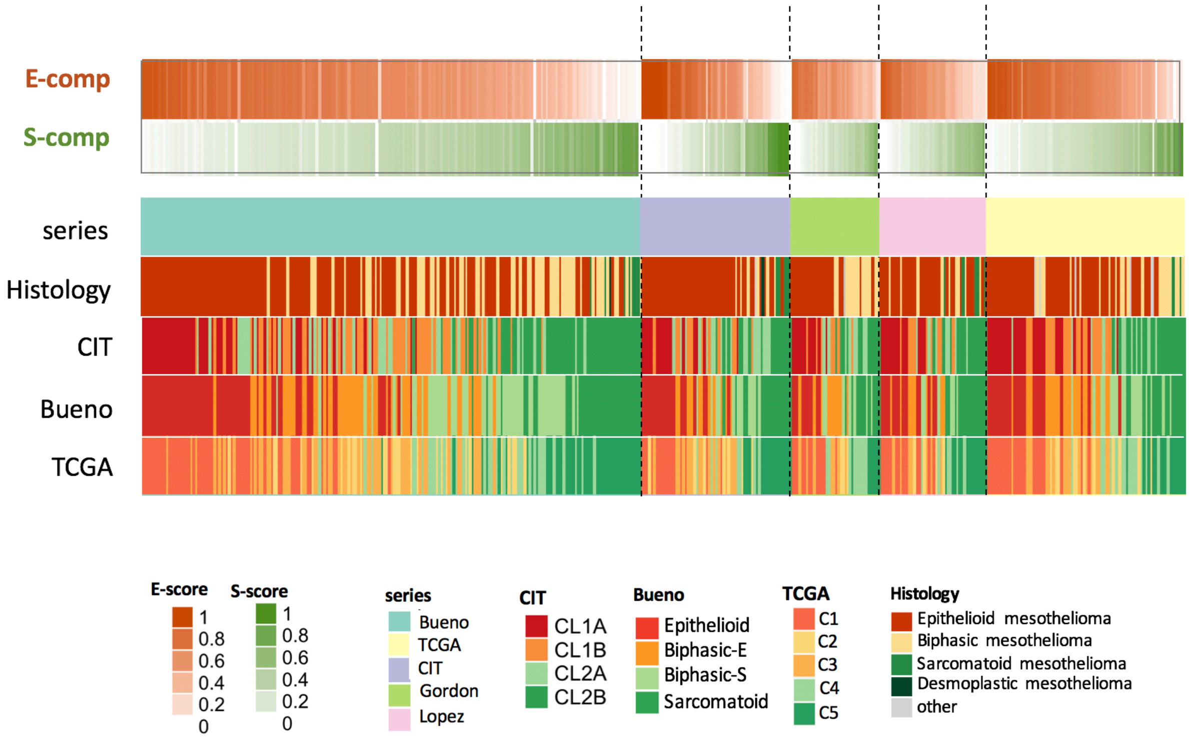

Supplementary Figure 4

Comparison of molecular gradients with histology and molecular subtype predictions of CIT, TCGA and Bueno classifications.

Estimation of the E-score and S-score in all available tumor tissue samples (442 samples). Samples were ordered by series and their E-score and S-score ratios.

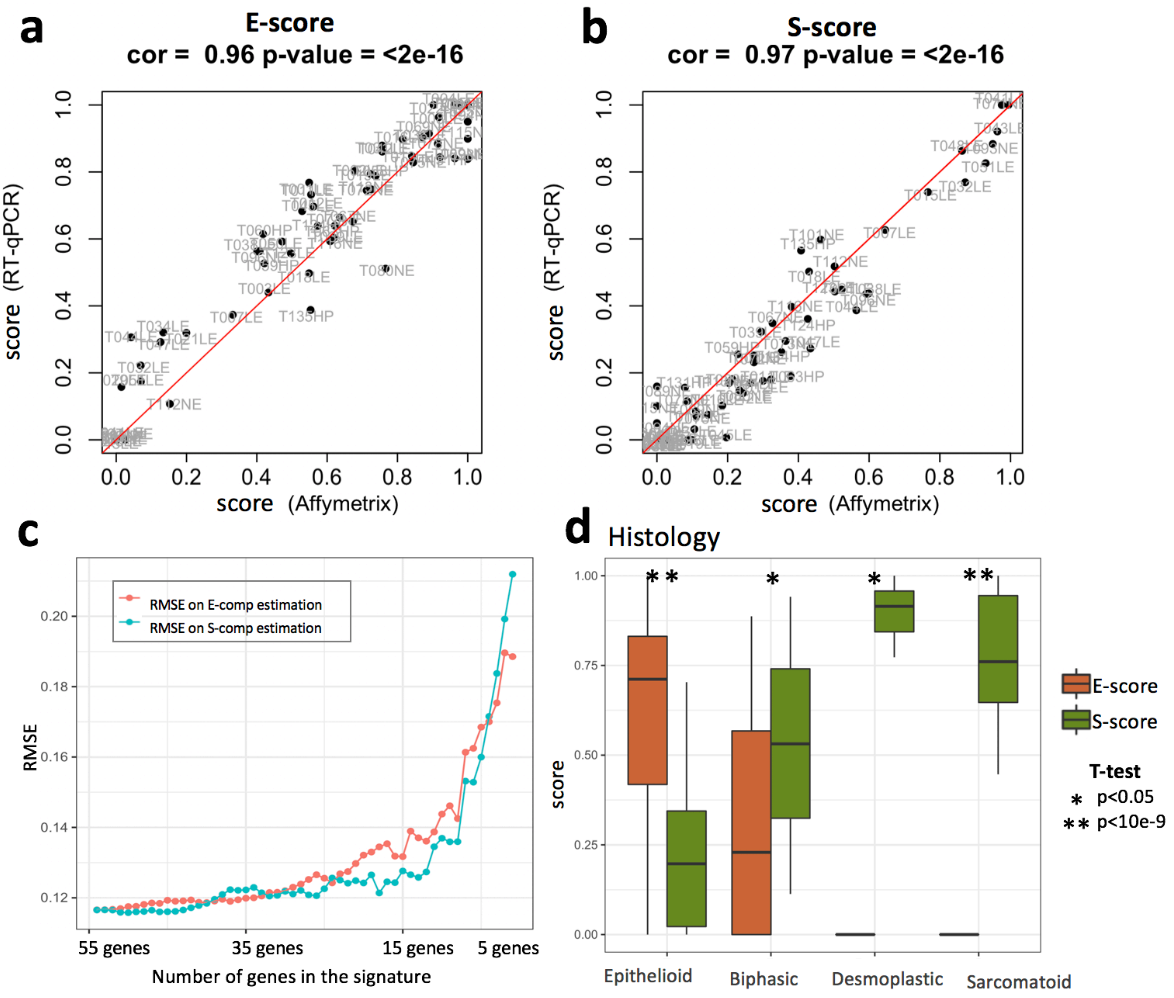

Supplementary Figure 5

Consistency between estimations on Affymetrix data and qRT-PCR data.

a,b Comparisons of the E-score (**a**) and the S-score (**b**) estimated using Affymetrix data or using qRT-PCR data in the CIT exploration series. Correlation coefficients and associated *P* values are shown. **c** The root mean squared error (RMSE) was calculated to measure the differences between the E-score and S-score estimated using affymetrix data (considered as the reference) or qRT-PCR data for a decreasing number of genes in the signature based on the CIT exploration series. Considering *P* value, the less significant gene was removed at each step. **d** Boxplot of E-score and S-score according to histology. Significance of t-test comparing E-score and S-score at each modality is shown (**P* value <0.05, ***P* value<10e-9,

NS: not significant). For all boxplots, bottom and top of boxes are the first and third quartiles of the data, respectively, and whiskers represent the lowest (respectively highest) data point still within 1.5 interquartile range of the lower (respectively upper) quartile. Center line represents the median value.

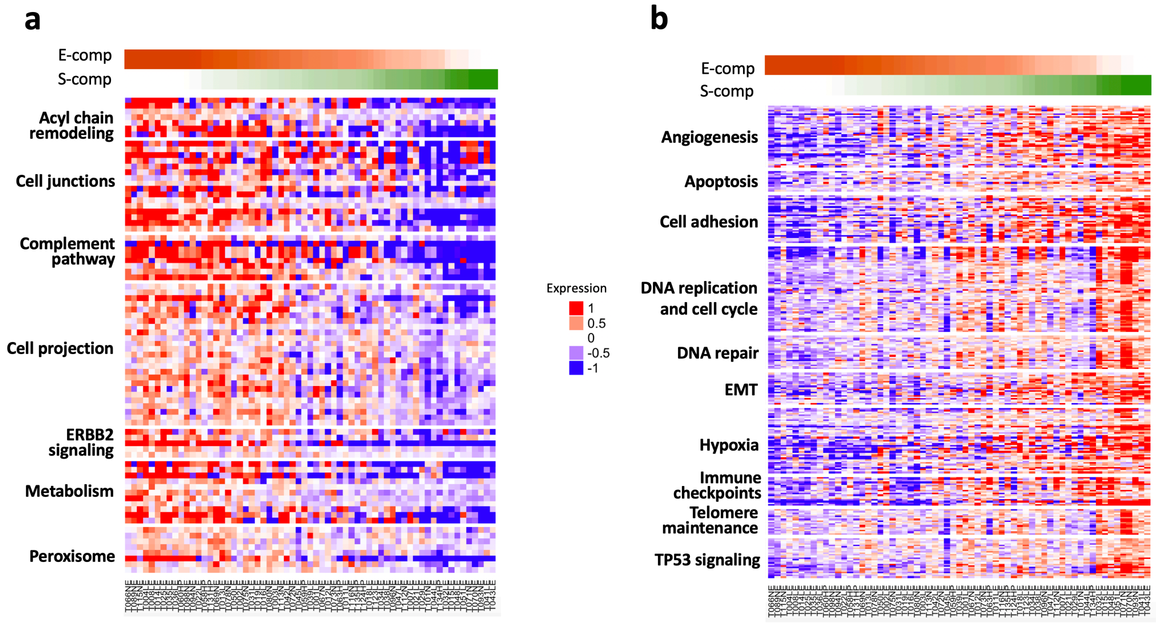

Supplementary Figure 6

Component-specific pathways associated to E-comp and S-comp.

Expression heatmaps at the gene level of the component-specific pathways (*P* value<0.05, Fisher's exact test) activated along E-comp (**a**) or S-comp (**b**). The names of the genes associated to the different pathways presented in **(a)** and **(b)** are given in Supplementary Table 3A and 3B respectively.

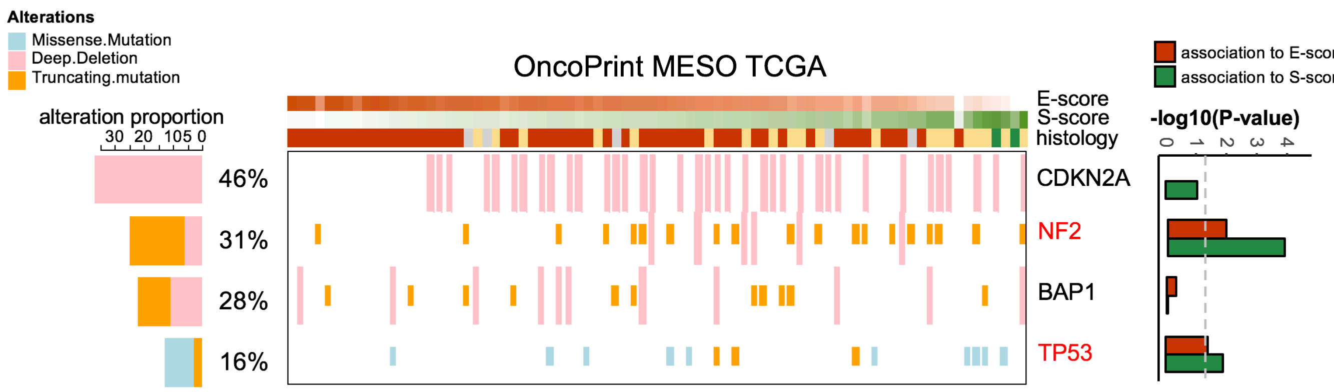

Supplementary Figure 7

Association between E-comp and/or S-comp and genetic alterations

Genetic alterations in well-known altered genes in MPM, including point mutations and copy number alterations (CNA) in the TCGA series. Lateral bars on the right correspond to -log10(*P* value) of the t.test comparing for a specific gene the E-score (brown) or the S-score (green) between samples with or without any alterations. The grey dashed line corresponds to a *P* value threshold of 0.05. Gene labels are colored in red when at least one of the corresponding tests is significant (*P* value<0.05).

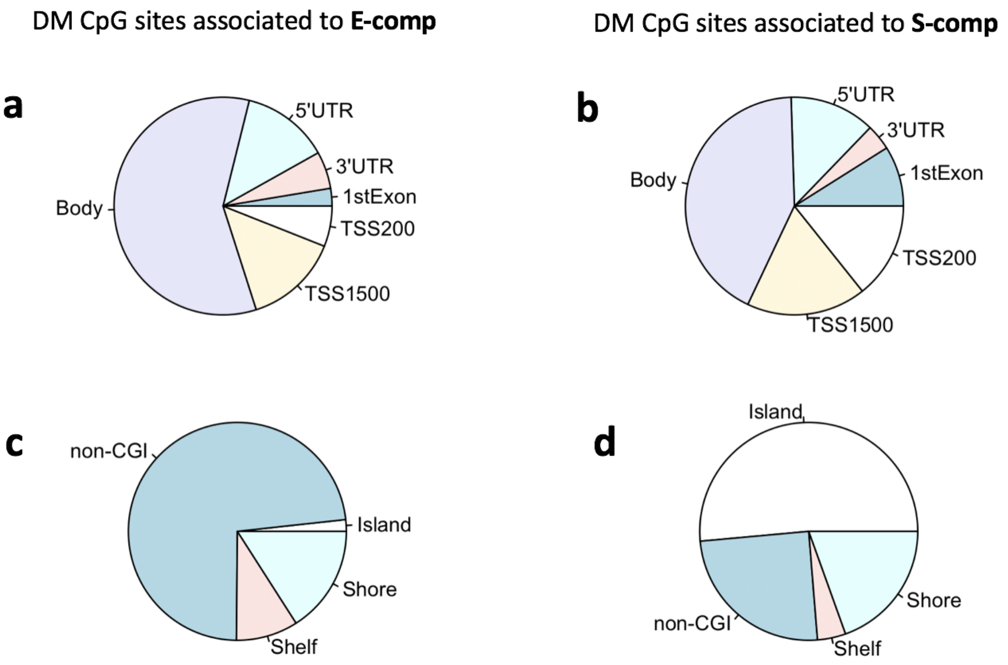

Supplementary Figure 8

Position characteristics of differentially methylated CpG sites associated to E-comp or S-comp.

a,**b** Pie charts showing the position frequency in genomic DNA region, including distance from transcriptional start site (TSS) and localization in untranslated transcription region (UTR) of mRNA, of the differentially methylated (DM) CpG sites associated to E-comp (**a**) or S-comp (**b**). **c**,**d** Position frequency in CpG islands (CGI), shores, shelves and non CpG Island (non CGI) of the differentially methylated CpG sites associated to E-comp (**c**) or S-comp (**d**).

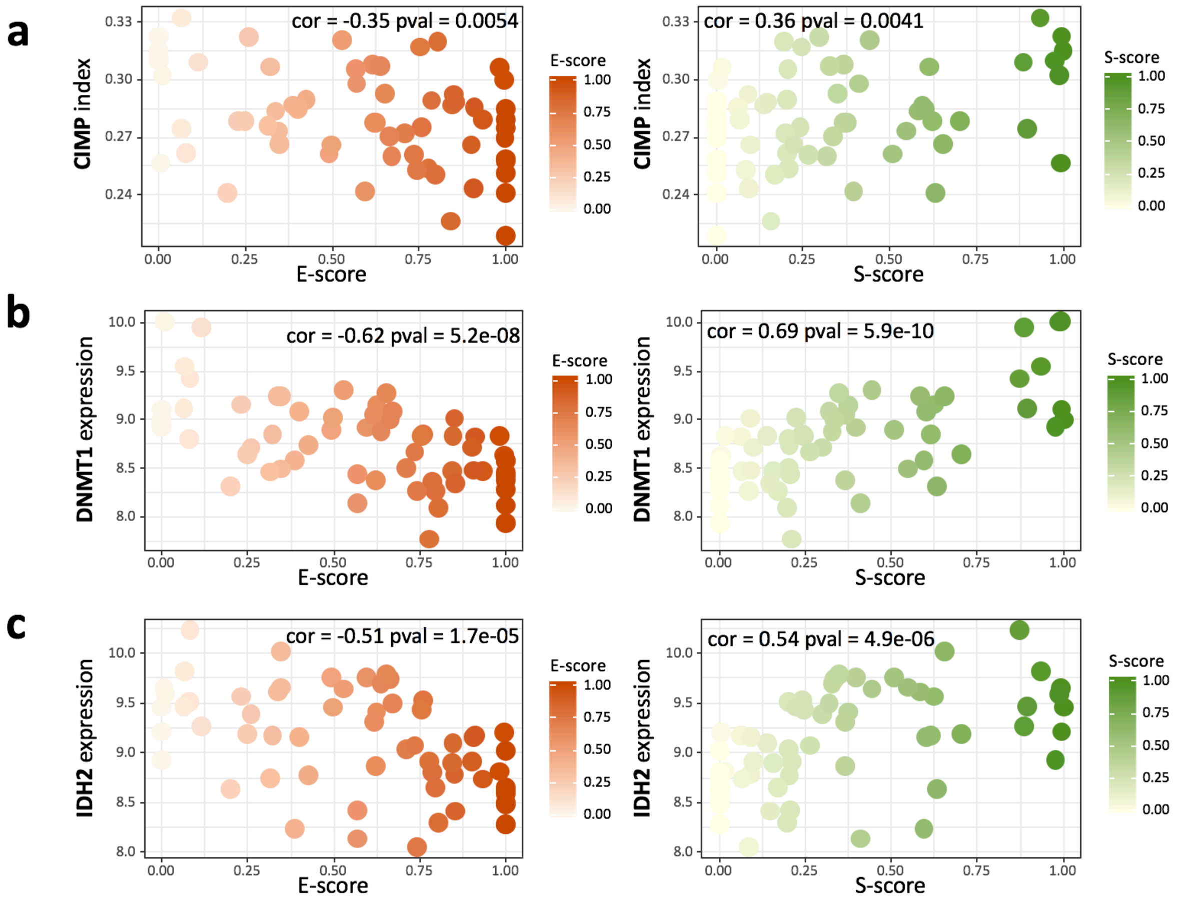

Supplementary Figure 9

E-comp and S-comp association to CpG Island hypermethylation and epigenetic regulators *DNMT1* and *IDH2*.

Correlation plots of CIMP score (**a**), *DNMT1* expression (**b**) or *IDH2* expression (**c**) and with the S-score or E-score. Color gradient changes correspond to the E-score and S-score values. For each plot, correlation coefficient and *P* value are shown (Pearson's correlation test).

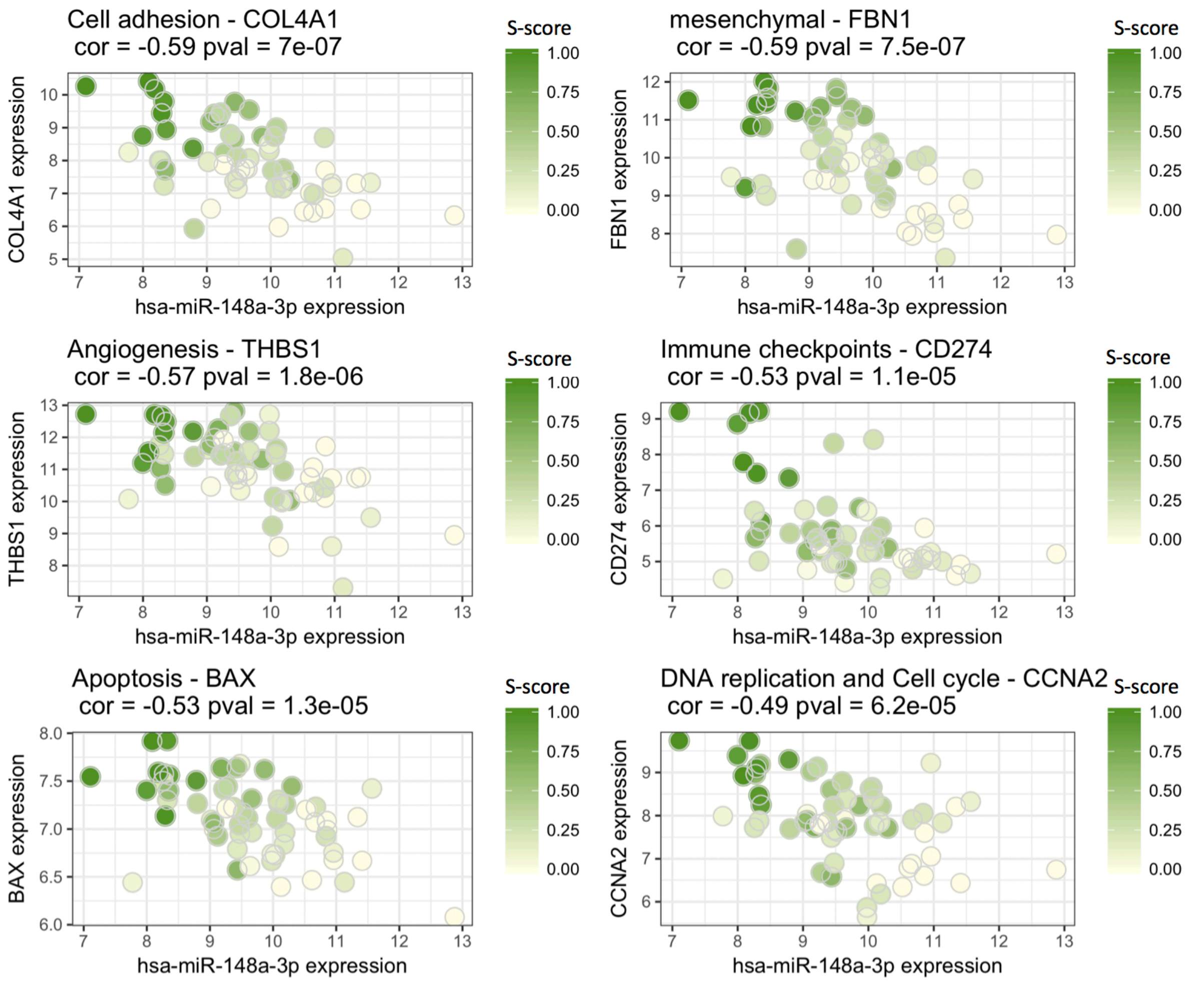

Supplementary Figure 10

Example of mir-148a-3p regulations.

Correlation plots between miR-148a-3p expression and several of its validated targets based on miRTarbase and TarBase databases.l Color gradient changes correspond to S-score. For each plot, correlation coefficient and *P* value are shown (Pearson correlation test).

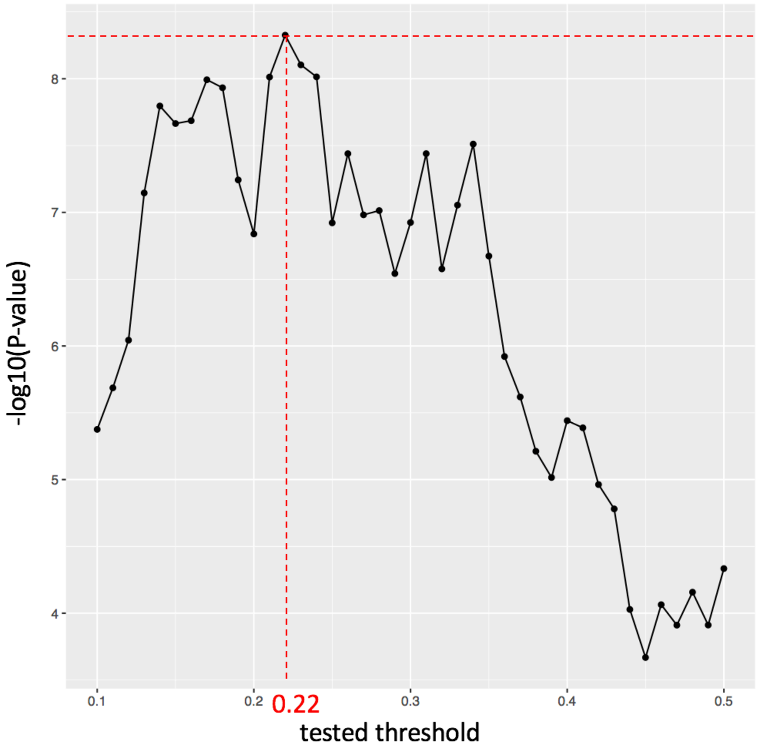

Supplementary Figure 11

Determination of the best survival discriminant threshold of the S-score.

For each tested threshold ranging from 0.1 to 0.5 with a step of 0.01, the average -log10(*P* value) over the 500 simulations is represented.

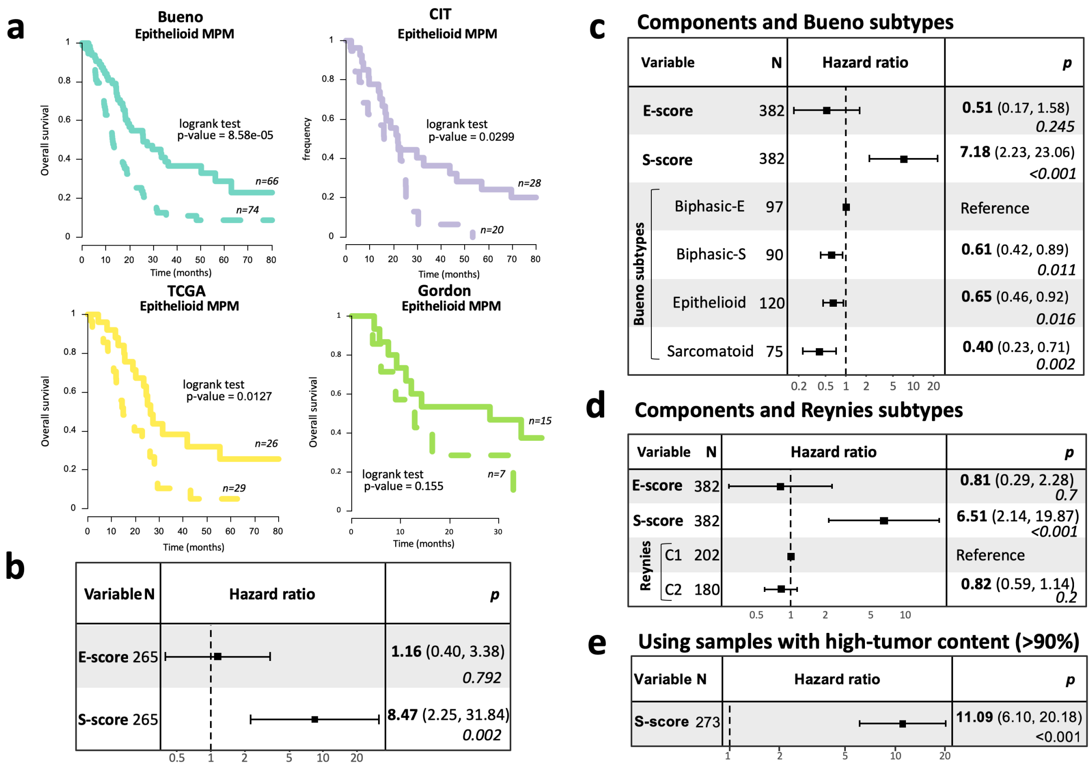

Supplementary Figure 12

Prognostic impact of the S-score.

a Overall survival curves plot for diagnosed epithelioid MPM patients with less than 20% of S-score (plain curve) or more than 22% of S-score (dashed curve) in the tumors of CIT, Bueno, TCGA and Gordon series. **b** Forest plots of overall survival hazard ratios (HRs) estimated by a multivariate Cox analysis adjusted for series, in epithelioid MPM patients. **c, d** Forest plots of overall survival HRs estimated by a multivariate Cox analysis adjusted for series, in epithelioid MPM patients integrating the E-score or S-score to Bueno (**c**) or Reynies subtypes (**d**). **e** Forest plots of overall survival hazard ratios (HRs) estimated by a multivariate Cox analysis adjusted for series, in MPM patients showing a tumor content higher than 90% (combined E-score and S-score).

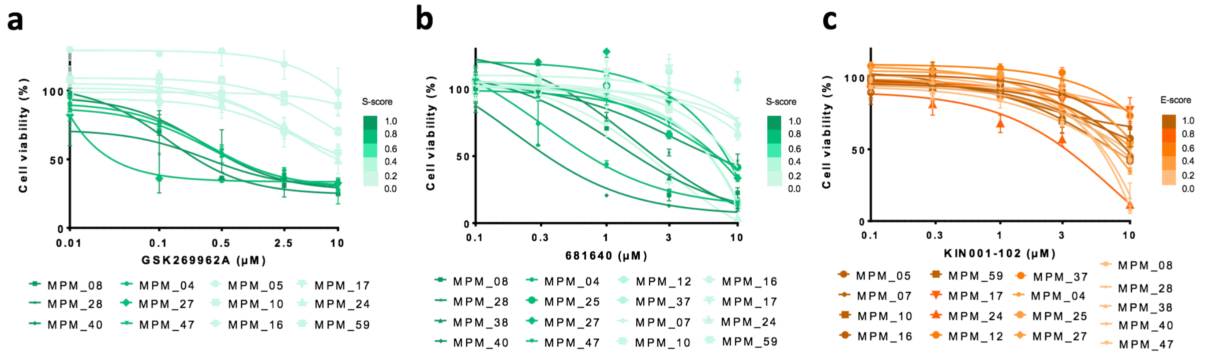

Supplementary Figure 13

Cell viability curves.

Cell viability was determined in the presence of a gradient concentration of GSK269962A ROCK inhibitor (**a**), 681640/Wee1 Inhibitor (**b**) and KIN001-102/Akt Inhibitor VIII (**c**) in 12 to 17 MPM in culture. Color gradient changes correspond to the E-score and S-score. Representative cell viability curves of one of the two independent experiments are shown. Each point represents the mean ± SD of triplicates.

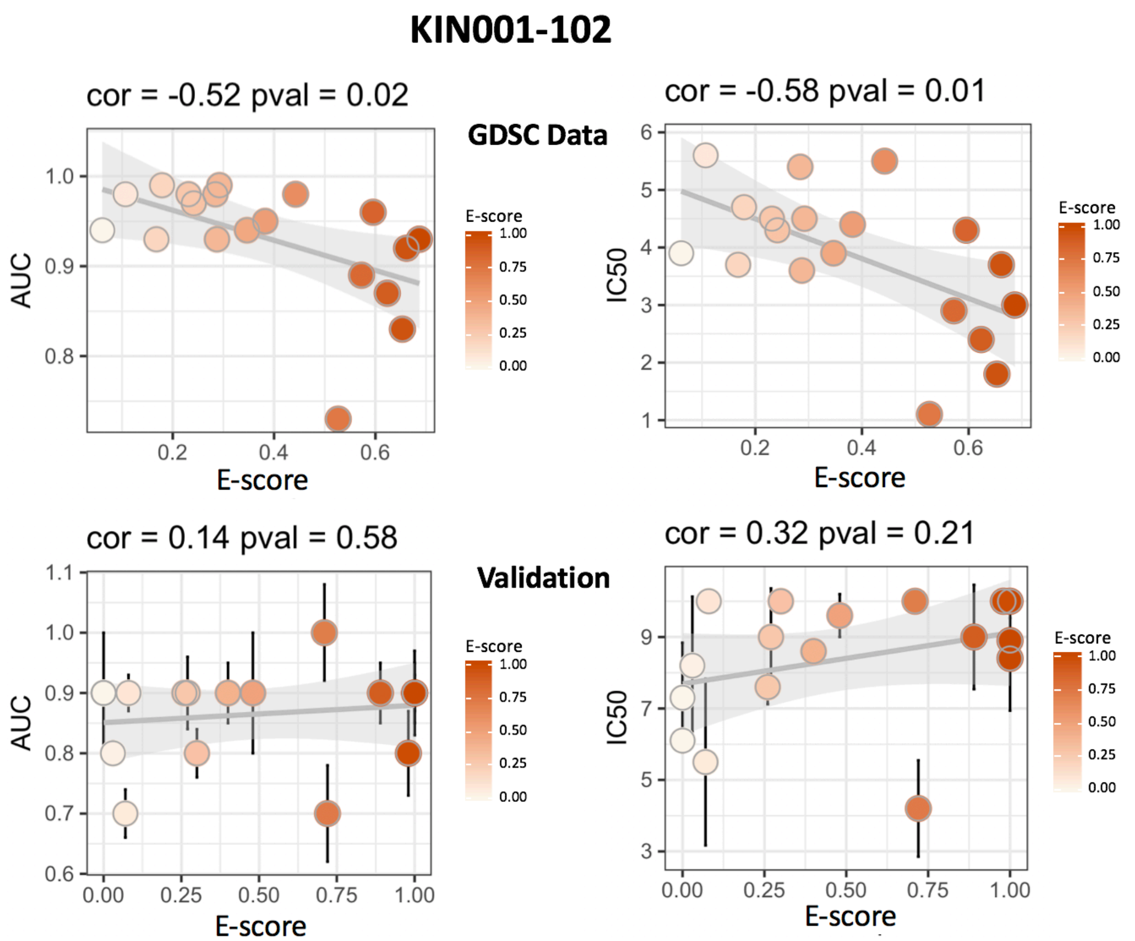

Supplementary Figure 14

Cell response to KIN001-102 with the E-score.

Correlation plots of AUC and IC50 for KIN001-102, obtained from GDSC data or determined from our validation experiments with the E-score. Color gradient changes correspond to the E-score. For each plot, correlation coefficient and *P* value are shown (Pearson's correlation test). Error bars correspond to s.d.

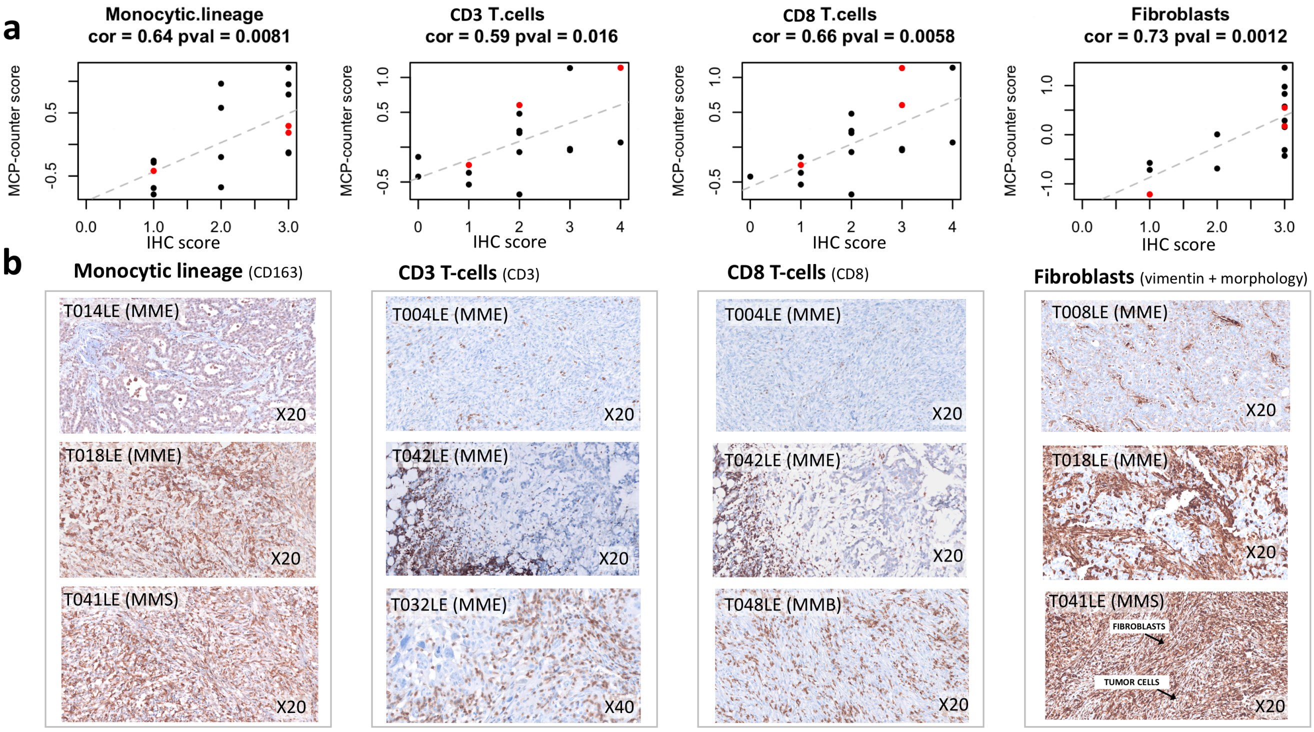

Supplementary Figure 15

Immunohistochemistry (IHC) on immune and stromal populations.

a Correlation between IHC staining scores and MCP-counter scores. Red dots correspond to samples for which corresponding immunostaining picture is shown. **b** Representative pictures of stained FFPE samples.

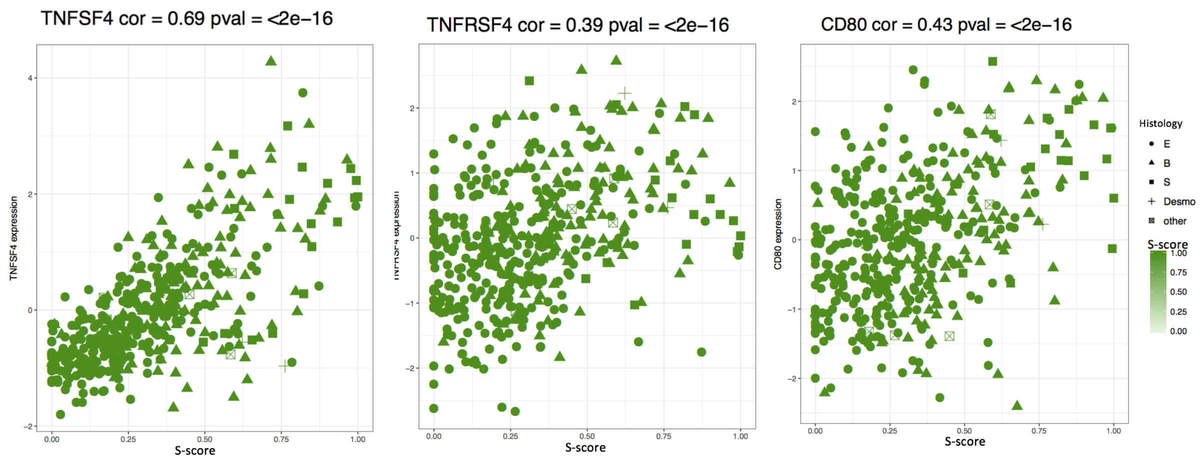

Supplementary Figure 16

Relation between immune checkpoints gene expression and the S-score.

Correlation plots of gene expression for different immune checkpoints (*TNFSF4*, *TNFRSF4 and CD80*) with the S-score. For each plot, correlation and *P* value are shown (Pearson's correlation test). Point shapes correspond to the different MPM histologies: Epithelioid (E), Biphasic (B), Sarcomatoid (S) and Desmoplastic (Desmo).

Supplementary Table 1

Antibodies used in immunohistochemical staining

Marker	Source	Clone	Dilution
**PD-L1**	DAKO	22C3	1:50
**CD163**	Biotech	K20-T	1:400
**CD3**	Leica	324944A	1:100
**CD8**	DAKO	C8/144	1:25
**Vimentin**	Ventana	V9	Prediluted
