# Supplementary material for: Dissecting heterogeneity in malignant pleural mesothelioma through histo-molecular gradients for clinical applications
Source: Nat Commun. 2019 Mar 22;10:1333. doi: 10.1038/s41467-019-09307-6 (PMC6430832; doi:10.1038/s41467-019-09307-6)
Supplement: Supplementary file 3 — Description of Additional Supplementary Files [file 41467_2019_9307_MOESM3_ESM.pdf]

Description of Additional Supplementary Files

File Name: Supplementary Data 1
Description: Excel workbook showing the expressions of selected genes determined by qRT-PCR (Taqman probe reference is indicated for each gene). Genes used in the qRT-PCR deconvolution signature are highlighted by the number 1 at the top of the gene name.

File Name: Supplementary Data 2
Description: Excel workbook listing the genes whose expression is positively correlated to the E-score or the S-score in separate spreadsheets.

File Name: Supplementary Data 3
Description: Excel workbook listing the pathways associated to the E-score or S-score. Details of the pathway enrichment analyses using different databases are given in separate spreadsheets. The two first excel spreadsheet tabs correspond to a summary of the selected pathways associated to the E-component and the S-component.

File Name: Supplementary Data 4
Description: Excel workbook listing CpG whose DNA methylation level is correlated to the E-score and/or the S-score.

File Name: Supplementary Data 5
Description: Excel workbook listing the miRNAs whose expression is correlated to the E-score and/or the S-score.

File Name: Supplementary Data 6
Description: Excel workbook giving the correlation and associated P-value between drugs sensitivity (AUC and IC50 determined using MPM cell lines and different drugs from the GDSC database) and the E-score and/or the S-score.

File Name: Supplementary Data 7
Description: Excel workbook giving the correlation and associated P-value between drugs sensitivity (AUC and IC50 determined using MPM cells in cultures established in Inserm UMRS-1138 laboratory and different drugs) and E-score and/or the S-score.

File Name: Supplementary Data 8
Description: Excel workbook giving the correlation between immune population score (MCPcounter) or ICK gene expression and the E-score or the S-score (spreadsheet tab A). and the correlation between ICK gene expression and T-cell MCPcounter score (spreadsheet tab B).

File Name: Supplementary Data 9
Description: Excel workbook giving the centroid of each subtype of the different classifications (CIT, Reynies, Bueno, TCGA, Gordon, Lopez) in separate spreadsheet tabs.

File Name: Supplementary Data 10

Description: Excel workbook giving the deconvolution signature for tissue samples or cell lines in separate spreadsheet tabs.

File Name: Supplementary Data 11
Description: Excel workbook giving the E-score and the S-score for each tumor sample in all the series (CIT, Reynies, Bueno, TCGA, Gordon, Lopez).
